# HIV-1 Tat Impairment of Mitochondrial Respiration via Complexes I and II Can Be Ameliorated by Allopregnanolone in Opioid-Exposed or Opioid-Naïve Cells and Mice

**DOI:** 10.3390/antiox14040420

**Published:** 2025-03-31

**Authors:** Fakhri Mahdi, Zia Shariat-Madar, Jason J. Paris

**Affiliations:** 1 Department of BioMolecular Sciences, School of Pharmacy, University of Mississippi, University, MS 38677-1848, USA; fmahdi@olemiss.edu (F.M.); madar@olemiss.edu (Z.S.-M.); 2 Research Institute of Pharmaceutical Sciences, University of Mississippi, University, MS 38677-1848, USA

**Keywords:** 5α-pregnan-3α-ol-20-one, electron transport chain, HIV, mitochondria function, mitoplast, drug interaction, trans-activating transcriptor, oxidative stress

## Abstract

HIV-associated neurocognitive disorders are prevalent despite antiretroviral intervention. Some HIV virotoxins, such as the trans-activator of transcription (Tat), are not targeted by antiretrovirals, and their neurotoxic actions may be exacerbated by opioids. Both Tat and morphine disrupt mitochondrial function, which may promote neurotoxicity, but the mechanisms are poorly understood. Herein, we assess the capacity of HIV Tat and morphine to alter the fundamental ability of mitochondria to generate and transfer energy along the electron transport chain (ETC). We find that exposure to Tat inhibits mitochondrial respiration driven by ETC complexes I or II in a concentration-dependent manner. Findings were consistent across models of permeabilized neuroblastoma cells, murine-derived mitoplasts, and mitochondria derived from mice exposed to Tat in vivo. In cell culture models, Tat promoted Ca^2+^ influx and the generation of cytosolic reactive oxygen species (ROS). Acute exposure to morphine exerted no effect on mitochondrial respiration, but morphine modestly offset Tat-mediated effects on complex I and some effects for the generation of ROS. Morphine did not exert any protective effects when acutely administered in vivo. The mitoprotective steroid, allopregnanolone (AlloP), increased mitochondrial respiration in neuroblastoma cells (complex I) or mitoplasts (complex II) and attenuated Tat-mediated impairment of complexes I and II in neuroblastoma cells or mice exposed to Tat in vivo. AlloP further attenuated Tat-mediated intracellular Ca^2+^ influx and cytosolic ROS production. Taken together, these results suggest that HIV Tat compromises mitochondrial function through the impairment of respiratory complexes I and II and that physiological AlloP may exert protective effects.

## 1. Introduction

People living with HIV (PLWH) continue to contend with HIV-associated neurocognitive disorders (HAND) despite antiretroviral interventions [1]. While the etiology of HAND is multifactorial, neurotoxic HIV proteins likely contribute. One of the most well-characterized HIV neurotoxic proteins is Tat, a key regulator of HIV replication and a soluble, cell-penetrating peptide that promotes neural cell damage and cell death. Current antiretroviral therapies do not target Tat, and it is detectable in the CSF of HIV patients, including those that are virally suppressed [2,3]. Preclinical evidence supports the notion that opioid exposure can further increase the neurotoxic effects of Tat [4,5,6]. However, such damaging effects on neurons appear to be driven in large part by proinflammatory and apoptotic factors that are downstream of glial activation, including innate immune hormones [7,8,9,10,11,12,13,14] and cargo carried by extracellular vesicles [14]. The molecular mechanisms whereby Tat and opioids may interact to disrupt neuronal function directly are not well-elucidated.

Mitochondria are a target of both Tat and opioids. Preclinical work demonstrates the mitotoxic capacity of Tat as evidenced by alterations in the polarization state of mitochondrial membranes [15,16], reduced oxygen consumption rate (OCR) [17] and ATP production [18], mitochondrial swelling, and the translocation of pro-apoptotic factors [19], as well as the production of ROS [20]. On the contrary, mitochondria express opioid receptors, and we have previously found morphine to offset Tat’s capacity to depolarize neuronal mitochondrial membrane potential [21], albeit morphine also exacerbates subsequent generation of Tat-mediated ROS in glia [21]. Some discrepancies exist in the extant literature with Tat either depolarizing [15,21] or hyperpolarizing mitochondrial membranes [16,21] and either depleting or increasing downstream ATP synthesis [22,23,24]. Differences in findings may be partly due to the early use of supraphysiological concentrations of Tat or differences in the models used. Greater use of more sensitive tools, such as the Seahorse analyzer, are contributing to a better understanding of Tat’s capacity to alter OCR. However, the primary mechanism(s) associated with mitochondrial dysfunction and cellular bioenergetic crisis begins with the fundamental inability of mitochondria to generate and transfer energy along the electron transport chain (ETC). Herein, we seek to identify the ETC complex(es) that are disrupted by Tat as well as to assess the potential interacting effects that may occur in the presence of morphine.

The impact of mitochondrial dysfunction on the central nervous system (CNS) extends beyond local effects for cellular bioenergetic crisis. The brain is a steroidogenic organ due to its capacity for *de novo* steroidogenesis (so-called ‘neurosteroidogenesis’) in neural cells, including neurons, astrocytes, microglia, and oligodendrocytes [25,26]. Not only are these cell types all demonstrated to be vulnerable to Tat-mediated toxicity, but they are also the source of *de novo* neurosteroids in the brain. We and others have demonstrated neurosteroids to exert a neuroprotective capacity in response to CNS insults, including Alzheimer’s disease [27,28] stroke [29,30], traumatic brain injury [31], and exposure to HIV Tat [21,32]. In particular, we have found that the 5α-reduced, 3α-hydroxylated metabolite of progesterone, allopregnanolone (AlloP), attenuates Tat-mediated neurotoxicity, astrogliosis, microgliosis, and behavioral deficits in Tat-transgenic mice [32]. Moreover, AlloP exerts known mitoprotectant effects, reducing the opening of the mitochondrial permeability transition pore (mPTP), increasing antiapoptotic Bcl-2 protein expression, and restoring basal mitochondrial membrane polarization states in response to insults including HIV Tat [21,33,34,35,36,37,38].

In this study, we investigated the effects of Tat on mitochondrial function under both native and morphine-exposed conditions. We further assessed the potential for the neurosteroid mitoprotectant, AlloP, to ameliorate Tat-mediated dysfunction. Having previously found AlloP or morphine to ameliorate Tat-induced mitochondrial depolarization in neurons [21], we hypothesized that Tat would disrupt the ETC and that this would be restored by physiological concentrations of AlloP and/or morphine. Moreover, we expected that cellular calcium influx and the subsequent generation of ROS by Tat would be attenuated by AlloP.

## 2. Materials and Methods

All experiments were pre-approved by the University of Mississippi Institutional Animal Care and Use Committee and were conducted in accordance with ethical guidelines defined by the National Institutes of Health (NIH Publication No. 85-23).

### 2.1. Animals and Housing

Timed-pregnant C57BL/6NHsd (C57BL/6N) or adult C57BL/6N mice (~50 days of age) were acquired from Envigo (Indianapolis, IN, USA). HIV-1 Tat transgenic mice (~60 days of age) were bred and maintained in the temperature- and humidity-controlled vivarium at the University of Mississippi (University, MS, USA). The generation of HIV-1 Tat_1–86_ transgenic mice (Tat-tg mice) has been previously described [39,40,41,42]. Expression of *tat_1__-86_* was confirmed by polymerase chain reaction (PCR) as described [42,43]. Mice were either singly housed (timed-pregnant C57BL/6N mice) or were housed four-to-five mice/cage (adult C57BL6/N or Tat-transgenic mice) and maintained on a reversed 12:12 h light cycle. Animals had free access to food and water *ad libitum* unless otherwise stated.

### 2.2. Chemicals

#### 2.2.1. Chemicals Used In Vitro and Ex Vivo

Cells were treated with vehicle or a saturating dose of morphine (500 nM in ddH_2_O; #M8777, Sigma-Aldrich, Saint Louis, MO, USA), vehicle or low-to-high physiological concentration of AlloP (0.1, 1, 10, or 100 nM dissolved in DMSO and diluted 1:10,000 in media; #P8887, Sigma-Aldrich, Saint Louis, MO, USA), and vehicle or a concentration–response curve of HIV-1 Tat_1–86_ (100, 200, 500, 600, or 800 nM diluted to concentration in ddH_2_O; #1002-2, ImmunoDx, Woburn, MA, USA). AlloP dosing reflects a range of physiological concentrations that have been found to confer protection from several neurodegenerative insults [38,44,45,46,47]. The Tat concentration–response curve includes high, non-physiological concentrations intended for mechanistic insights and alignment with prior reports that have used such concentrations. The primary concentration of Tat used (100 nM) reflects one from a range that elicits functional deficits in glia and neurons similar to those observed in HIV infection [48,49,50,51,52,53].

#### 2.2.2. Chemicals Used In Vivo

Mice were treated with vehicle or morphine (30 mg/kg, i.p., dissolved in sterile 0.9% saline; #M8777, Sigma-Aldrich, Saint Louis, MO, USA) as well as vehicle or AlloP (1 mg/kg, s.c., dissolved in EtOH and diluted 1:10 in oil; #P0130, Sigma-Aldrich, Saint Louis, MO, USA). To induce HIV-1 *tat_1–86_* transgene expression (or not), Tat(−) and Tat(+) control mice were induced via injection of doxycycline (30 mg/kg, i.p.; #14422, Cayman Chemical, Ann Arbor, MI, USA), followed by two days washout. The chosen morphine concentration is demonstrated to enhance AlloP in the rodent brain and circulation when administered acutely [54,55].

### 2.3. Assessment of Mitochondrial Respiration

#### 2.3.1. Mitochondria Derived from SH-SY5Y Neuroblastoma Cells

Human SH-SY5Y neuroblastoma cells (obtained from ATCC, #CRL-2266; Manassas, VA, USA) were seeded onto a T-225 flask at a density of 5 × 10^6^ cells/flask. Cells were maintained in growth medium: 89.5% DMEM/F12 (Life Technologies, Carlsbad, CA, USA), 10% heat-inactivated fetal bovine serum (FBS; Thermo Scientific Hyclone, Logan, UT, USA), and 0.5% antibiotic/antimycotic mixture (Life Technologies, Carlsbad, CA, USA). The OCR in human SH-SY5Y neuroblastoma cells was determined via an Oxytherm Clarke-type electrode system (Hansatech, Germany, distributed by PP System, MA, USA) and was used to assess the effects of HIV-1 Tat_1–86_, AlloP, and/or morphine. SH-SY5Y cells were trypsinized and centrifugated at 180× *g* for 5 min at room temperature, and the pellet was washed once with DPBS. The final cell pellet was re-suspended in DPBS to a concentration of 200 × 10^6^ cells/mL. Studies were performed on mitochondria with the plasma membrane permeabilized by digitonin (final concentration of 5 μm). A volume of 100 μL of the cell suspension (20 × 10^6^ cells/run) was added to the chamber of the Oxytherm Clark-type electrode system containing 0.9 mL of mitochondria buffer (20 mM HEPES, pH 7.3, 120 mM KCl, 2 mM KH_2_PO_4_, 2 mM MgCl_2_, 1 mM EGTA) which was equilibrated at 37 °C. Once baseline respiration was established, the compounds were injected into the chamber at 3-min intervals using a Hamilton syringe. The mitochondria buffer was supplemented with 5 mM sodium pyruvate and sodium malate (Sigma-Aldrich, Saint Louis, MO, USA) to energize complex I, 5 mM sodium succinate dibasic hexahydrate (Sigma-Aldrich, Saint Louis, MO, USA) to energize complex II, and 5 mM l-ascorbic acid plus 0.2 mM *N*, *N*, *N*′, *N*′-tetramethyl-p-phenylenediamine for complex IV (See Supplemental Figure S1A–C). The use of these substrates was necessary to provide electrons to different sites within the mitochondrial ETC. The integrity of mitochondria was evaluated by using known complex I and III inhibitors, rotenone (Sigma, Saint Louis, MO, USA) and antimycin A (Sigma, Saint Louis, MO, USA), respectively (Supplemental Figure S1A,B). The inhibitors were added at a final concentration of 1 μm. The final concentration of solvent used was less than 0.1% (*v*/*v*). Neither Tat (Supplemental Figure S1C), morphine (Supplemental Figure S1E), nor AlloP (Supplemental Figure S1F) prevented mitochondrial respiration from responding to complex activators or inhibitors.

#### 2.3.2. Mitochondria Derived from Mouse Brain

Mitochondria were derived from C57BL/6NHSd mice or from Tat-tg mice that carried the *tat* transgene [Tat(+)] or did not carry the transgene [Tat(−)] (*n* = 2 brains per experimental replicate). Mitochondria from mouse brains were isolated using the high-yield, differential centrifugation procedure (Bio-Protocol LLC, Sunnyvale, CA, USA; https://bio-protocol.org, accessed 1 February 2021). Mice were fasted for 16 h overnight (i.e., the last 4 h of their dark cycle and all 12 h of their light cycle). Mice were sacrificed by cervical dislocation. When Tat(−) or Tat(+) mice were used, the mice were first treated with AlloP (1 mg/kg, s.c., QD) for 7 days while *tat* expression was contemporaneously induced via doxycycline (30 mg/kg, i.p., QD for 5 days, followed by 2 days of washout [56,57]; with or without morphine (30 mg/kg, i.p.) administered 60 min prior to tissue collection and mitochondria preparation on day 8. It is notable that chronic exposure to doxycycline can injure mitochondria, but the current dosing was low, and the exposure was very acute. All mitochondrial preparations demonstrated the expected yields and respiratory capacity as described. Mouse brains were isolated and placed in cold extraction buffer (1.25 mM Sucrose, 250 mM mannitol, 10 mM HEPES, and 10 mM EGTA) supplemented with freshly added 0.01% BSA (Sigma, MO) and 1× protease inhibitor (Fisher Scientific, IL). After mincing and homogenizing the brain(s) with a Dounce homogenizer (grind the brain 20 times) on ice, the homogenate was centrifugated at 700× *g* for 10 min at 4 °C. The supernatant was re-centrifugated at the same speed for an additional 10 min at 4 °C. The supernatant of the second centrifugation was centrifugated at 10,000× *g* for 15 min at 4 °C. The pellet was re-suspended in small volume, and protein was measured at 280 nm using a NanoDrop 2000c Spectrophotometer (Thermo Scientific, Waltham, MA, USA). Stock suspension(s) containing 3 mg of proteins/mL were stored on ice in an extraction buffer without BSA and protease inhibitor. The yield for mitochondria averaged 3.95 mg/mL for every two brains (SEM = 0.04). Mitochondria (230 μg) were assessed for respiration as described above.

#### 2.3.3. Mitoplasts Derived from Mouse Brain

To derive mitoplasts (*n* = 2 brains used per experimental replicate), the mitochondrial pellets were re-suspended in extraction buffer, supplemented with 0.01% BSA and protease inhibitor, containing 0.02% digitonin (*w*/*v*) for 10 min on ice, followed by centrifugation at 10,000× *g* for 15 min at 4 °C. Like mitochondria, the mitoplast pellet was also re-suspended in extraction buffer without BSA or protease inhibitor and protein concentration was measured at 280 nm using a NanoDrop 2000c Spectrophotometer. The yield for mitoplasts averaged 3.14 mg/mL for every two brains (SEM = 0.04). Mitoplasts (230 μg) were assessed for respiration as described above.

### 2.4. Assessment of Intracellular Calcium

SH-SY5Y neuroblastoma cells were seeded on 35 mm glass-bottom dishes (MatTek, Ashland, MA, USA) and differentiated via sequential exposure to retinoic acid (3.33 mM) and BDNF (1.85 μM) as previously-described [58]. For imaging of intracellular calcium [Ca^2+^]_i_, cells were loaded with fura-2 AM (2.5 μm, Invitrogen, Carlsbad, CA, USA) for 20 min (37 °C, 5% CO_2_), then washed once and incubated in media for another 20 min. The second incubation allows for the de-esterification of the acetoxy methylester (AM) group. Ratiometric fluorescent imaging (λex: 340 and 380 nm; λem: 510 nm) was conducted such that one image per second was collected for the first 120 s, then one image every 30 s was collected for the next 480 s, and then one image every 60 s was collected for the last 600 s (20 min total). For analysis, three regions of interest were randomly selected on the cytoplasm of each cell. The mean [Ca^2+^]_i_ was calculated from the average F340/F380 ratio using a standard curve generated by a calcium calibration buffer kit (Life Technologies, Carlsbad, CA, USA) as previously described [59].

### 2.5. Assessment of Oxidative Stress

#### 2.5.1. Nuclear and Cytosolic Oxidative Stress via CellROX

Primary neurons were isolated from C57BL/6N mice as previously described [59] and were seeded onto 24-well plates at a density of 7.5 × 10^4^/well in neuron media containing glucose, NaHCO_3_, glutamine/glutamate, and B-27 supplement (Fisher Scientific, Hanover Park, IL, USA) at 37 °C with 5% CO_2_. The media was changed after 24 h and, for a longer period of cultivation, replaced 1:1 with fresh media every 3 days. After 8 days of culture, the neurons were treated with Tat (100 nM) in the absence or the presence of AlloP (10 or 100 nM) and/or morphine (500 nM) and incubated at 37 °C with 5% CO_2_ for 24 h. At the end of the incubation, the neurons were washed with DPBS, and CellROX reagent (Invitrogen, Waltham, MA, USA) was added at a final concentration of 5 µM for 30 min at 37 °C to detect ROS generation in the cytosol or nucleus per manufacturer’s instructions. The cells were washed twice with DPBS and maintained in 1 mL of HBSS. Images were collected using a Nikon Ti-E microscopy system (Melville, NY, USA) with a motorized stage. The fluorescent intensity of CellROX orange (cytosolic) and green (nuclear) signals were analyzed using ImageJ FIJI [60].

#### 2.5.2. Assessment of ROS via CM-H_2_DCFDA

ROS was also assessed in SH-SY5Y cells and primary mixed glia by 5- (and-6)-chloromethyl-2′,7′-dichlorodihydrofluorescein diacetate, acetyl ester (CM-H_2_DCFDA, Invitrogen, Carlsbad, CA, USA). SH-SY5Y cells were seeded at 2.5 × 10^4^/well in a 96-well plate and differentiated as previously described [58]. Primary mixed glia were isolated from timed-pregnant C5BL/6N mice at 14–17 days of gestation, and glia were seeded at 22 × 10^4^/well in a 96-well plate [61]. SH-SY5Y or mixed glial cells were treated with vehicle, Tat, AlloP, and/or morphine and incubated for 20 h (37 °C with 5% CO_2_). Cells were then loaded with CM-H_2_DCFDA (10 μM) for 60 min per the manufacturer’s protocol, washed twice, and measured (λex = 485; λem = 528 nm) via a CLARIOstar microplate reader (BMG Labtech, Inc., Cary, NC, USA). Arbitrary fluorescent units were calculated as the percentage change from media-treated control wells on each plate.

### 2.6. Statistical Analyses

**Figure 1:**
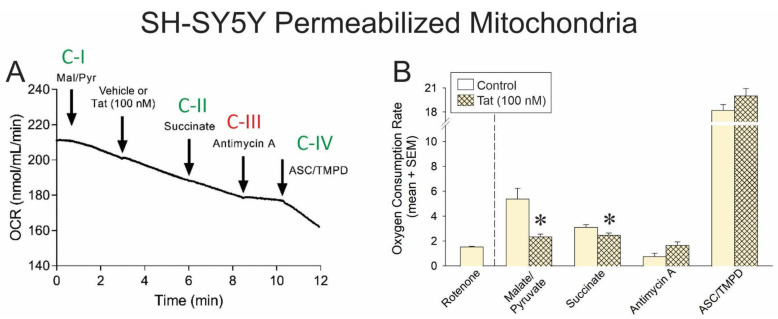
(**A**) Oxygen consumption rate (OCR) of permeabilized SH-SY5Y human neuroblastoma cells (n = 3–11 independent cultures/group) was assessed via a Clark-type electrode (representative oxygraph shown). Cells were sequentially exposed to pharmacological activators of complex I (malate and pyruvate), an activator of complex II (succinate), an inhibitor of complex III (antimycin A), activators of complex IV (ascorbate and TMPD), and a complex I inhibitor (rotenone). Downward arrow indicates onset of reagent effect. (**B**) The OCR of control cells was compared to that of cells pretreated with HIV Tat (100 nM) within each pharmacological condition (* indicates significant difference from respective control; two-tailed Student’s *t*-test, *p* < 0.05). Arrows indicates addition of reagent.

**Figure 2:**
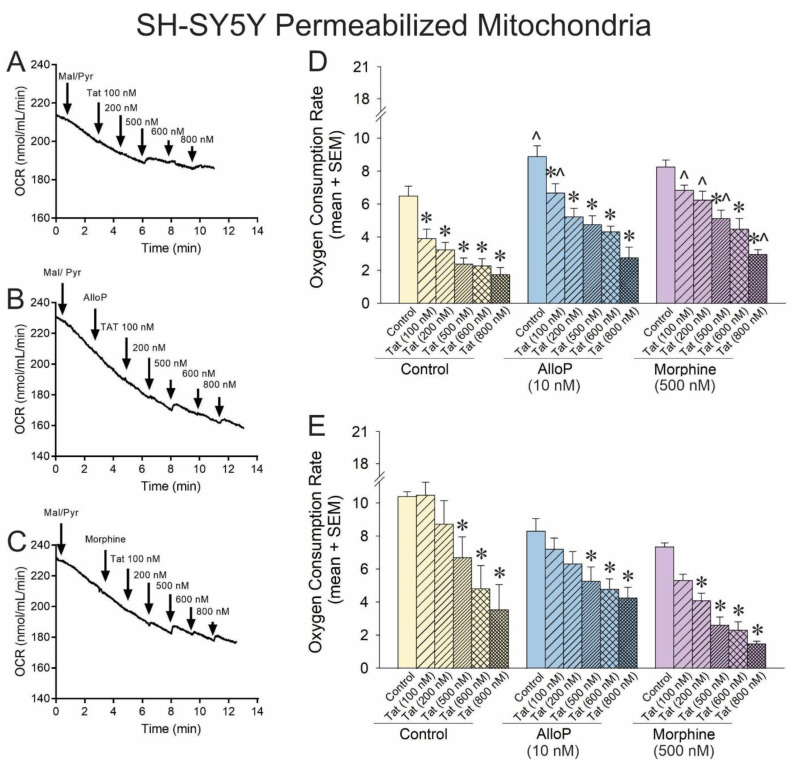
OCR was assessed for permeabilized SH-SY5Y human neuroblastoma cells exposed to a concentration–response curve of HIV Tat (100–800 nM; n = 3–15 independent cultures/group). Representative oxygraphs are shown for (**A**) control pretreatment, (**B**) pretreatment with allopregnanolone (AlloP; 10 nM), or (**C**) pretreatment with morphine (500 nM). (**D**) When mitochondrial respiration was driven from complex-I, Tat causedcaused a concentration-dependent decreasedecrease in the maximal level of OCR in SH-SY5Y cells, and AlloP or morphine offset this effect [*F*(17,74) = 9.95, *p *< 0.05]. (**E**) When mitochondrial respiration was driven from complex-II, neither AlloP nor morphine influenced the concentration-dependent reduction in OCR by Tat [*F*(16,55) = 6.49, *p* < 0.05] (* indicates significant reduction in OCR compared to respective control; ^ indicates significant increase in OCR with AlloP- or morphine-pretreatment compared to the same Tat concentration in the control group; one-way ANOVA, *p* < 0.05). Arrows indicates additionDownward arrow depicts onset of reagent effect.

**Figure 3:**
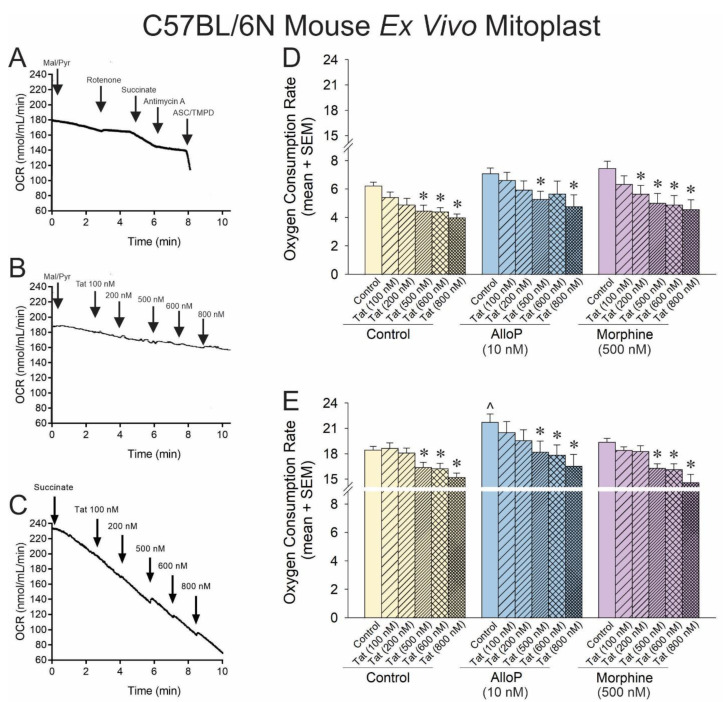
OCR was assessed for neural mitoplasts derived from C57BL/6N mice (n = 5–21 independent cultures/group). Representative oxygraphs are shown for (**A**) pharmacological activation and inhibition of complexes I–IV of the electron transport chain and the effects of Tat (100 nM) on (**B**) complex-I-mediated mitochondrial respiration or (**C**) complex-II-mediated mitochondrial respiration. (**D**) When respiration was driven from complex I, Tat reduced OCR in a concentration-dependent fashion, and neither AlloP nor morphine offset this effect [*F*(17,87) = 2.87, *p* < 0.05]. (**E**) When respiration was driven from complex-II, AlloP increased baseline OCR, but neither AlloP nor morphine influenced Tat-mediated effects to reduce OCR [*F*(17,93) = 4.68, *p* < 0.05] (* indicates significant reduction in OCR compared to respective control; ^ indicates significant increase in OCR with AlloP-pretreatment compared to the baseline control group; repeated measures ANOVA, *p* < 0.05). Arrows indicates addition of reagent.

**Figure 4:**
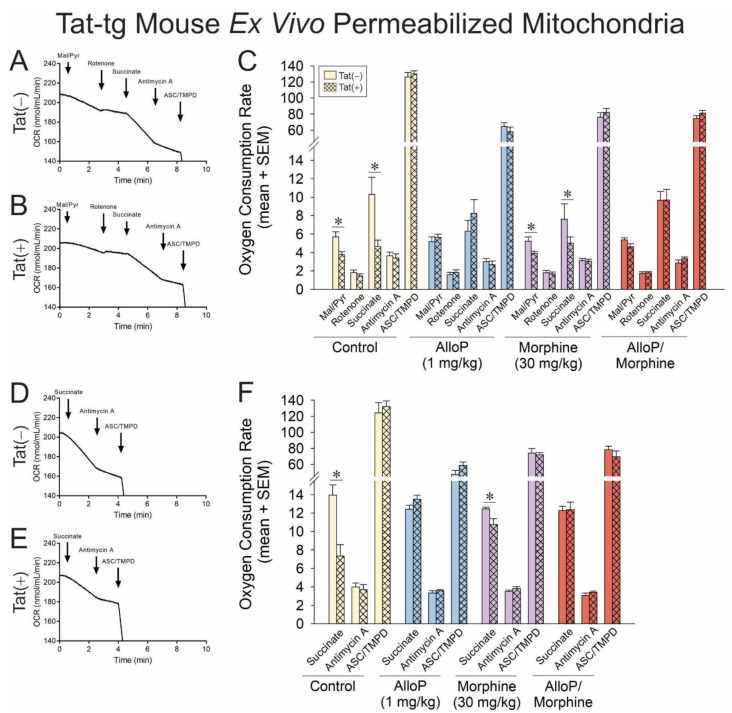
OCR was assessed for permeabilized neural mitochondria that were derived from transgenic mice that expressed HIV Tat in the brain (Tat^+^) or did not (Tat^−^). Some mice were administered AlloP (1 mg/kg, QD for 7 days) and/or subacute morphine (10 mg/kg, QD once) prior to tissue collection (n = 3–9). Representative oxygraphs are shown for complex I-mediated mitochondrial respiration in (**A**) Tat^−^ or (**B**) Tat^+^ mice and complex II-mediated mitochondrial respiration in (**C**) Tat^−^ or (**D**) Tat^+^ mice. The OCR of (**E**) complex-I- or (**F**) complex-II-driven mitochondrial respiration is shown in response to pharmacological activators and inhibitors of complexes I-IV (* indicates significant difference from respective control; two-tailed Student’s *t*-test, *p* < 0.05). ArrowsDownward arrow indicates additiononset of reagent effect.

**Figure 5:**
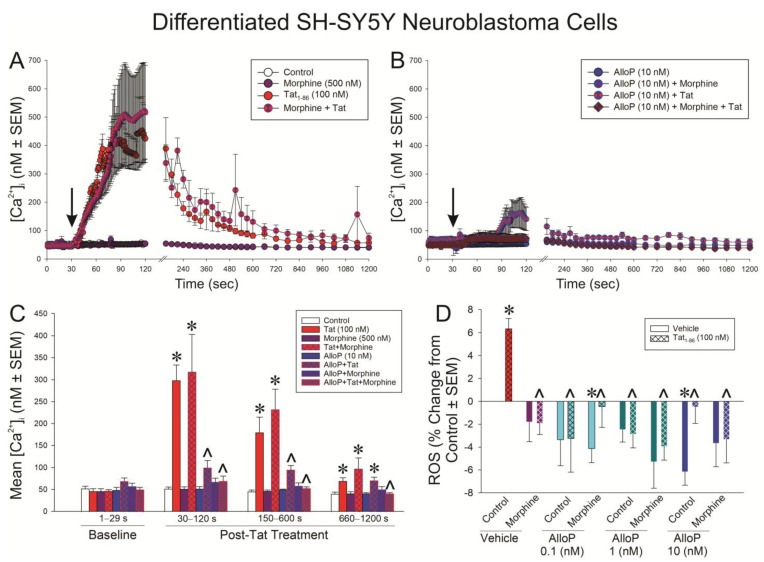
Differentiated SH-SY5Y neuroblastoma cells were assessed for (**A**,**B**) intracellular calcium [Ca^2+^]_i_ (n = 3–6 independent cultures/group; analyzed in panel (**C**) [*F*(21,60) = 14.32, *p* < 0.05]) or (**D**) reactive oxygen species (ROS) formation (n = 8 independent cultures/group; [*F*(15,112) = 2.91, *p* < 0.05]) following exposure to Tat (100 nM), AlloP (0.1, 1, or 10 nM), and/or morphine (500 nM). * indicates significant difference from respective control; ^ indicates significant reduction from respective Tat-treated group; repeated measures ANOVA in (**C**) and one-way ANOVA in (**D**) (*p* < 0.05). Arrow in (**A**,**B**) indicates the time of vehicle (ddH_2_O) or Tat administration.

**Figure 6:**
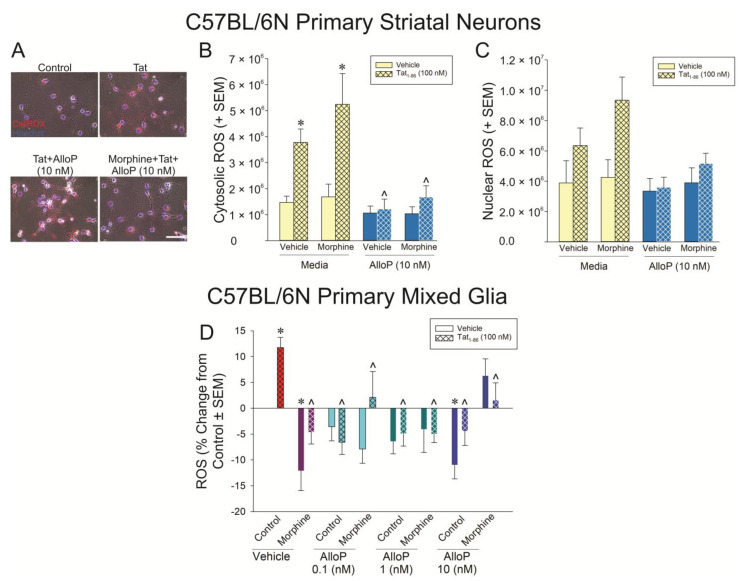
Striatal neurons (**A**–**C**; n = 4 independent cultures/group) or mixed glia (**D**; n = 10 independent cultures/group) were derived from C57BL/6N mice and assessed for (**A**,**B**) cytosolic neuronal reactive oxygen species (ROS) formation [*F_Morphine Cond_*(1,24) = 1.09, *p* < 0.05; *F_AlloP x Tat Cond_*(1,24) = 7.58, *p* < 0.05], (**C**) nuclear neuronal ROS formation, or (**D**) glial ROS formation [*F*(3,144) = 3.01, *p* < 0.05] following exposure to Tat (100 nM), AlloP (0.1, 1, or 10 nM), and/or morphine (500 nM). * indicates significant difference from respective control; ^ indicates significant reduction from respective Tat-treated group; three-way ANOVA in (**B**) and one-way ANOVA in (**D**), *p* < 0.05). Scale bar = 50 μm.

Assessments of mitochondrial respiration were analyzed via Student’s two-tailed, independent *t*-test (Figure 1), one-way analyses of variance (ANOVAs; Figures 2 and 3), or Student’s two-tailed, independent *t*-test (Figure 4) Assessments of [Ca^2+^]_i_ were analyzed via repeated measures ANOVA (Figure 5). Assessments of SH-SY5Y ROS (Figure 5) and glial ROS (Figure 6) were analyzed via one-way ANOVAs. Assessments of primary neuronal ROS were analyzed via three-way ANOVAs (Figure 6). Main effects were followed by Fisher’s Protected Least Significant Difference post hoc tests to determine group differences. Interactions were delineated via a priori planned main effect contrasts. All analyses were considered significant when *p* < 0.05 with α controlled for family-wise error.

## 3. Results

### 3.1. Tat Impairs Mitochondrial Respiration via Actions at Complexes I and II in SH-SY5Y Cells

In order to identify the important complexes of the ETC that HIV Tat interacts with to attenuate mitochondrial respiration, we assessed the OCR of human SH-SY5Y neuroblastoma cells that were digitonin-permeabilized and exposed to sequential activators of complex I (malate and pyruvate), complex II (succinate), an inhibitor of complex III (antimycin A), and activators of complex IV (ascorbate and TMPD; Figure 1A). After all other interventions, a complex I inhibitor (rotenone) was included with each replicate as a control to ensure that mitochondria were intact and respiring after pharmacological manipulation (Figure 1B, left of hatched line).

In control cells, malate and pyruvate drove complex I-mediated OCR (Figure 1B, open bars), and the addition of Tat significantly impaired complex I-mediated oxygen (O_2_) consumption (Figure 1B; hatched bar, see *). Similarly, the addition of succinate drove complex II-mediated OCR (Figure 1B, open bars), but in cells that were Tat-exposed, a significant (albeit modest) reduction in O_2_ consumption was observed (Figure 1B; hatched bar, see *). No significant differences were observed in the effect of complex III inhibition or complex IV activation between vehicle- or Tat-exposed cells. Notably, cells were also able to achieve apparent maximal respiration in response to FCCP (an H^+^ ionophore and uncoupler of oxidative phosphorylation) following an inhibitor of complex V (i.e., ATP synthase) and exposure to Tat (Supplemental Figure S1D). Together, these data support the notion that Tat’s effects are constrained to complexes I and II in this model.

### 3.2. AlloP or Morphine Attenuate Tat’s Capacity to Impair Complex I-Mediated Respiration

To assess the degree of inhibition that HIV Tat exerts over complexes I or II, SH-SY5Y cellular respiration was induced by complex I (malate and pyruvate) or complex II (succinate) activators, and cells were exposed to a concentration–response gradient of Tat protein (Figure 2A). To further assess the capacity of AlloP or morphine to modulate these effects, some cells were pretreated with AlloP (Figure 2B) or morphine (Figure 2C) prior to the application of Tat.

Complex I-mediated OCR was significantly impaired by any concentration of Tat (Figure 2D; yellow hatched bars, see *). Conversely, exposure to AlloP significantly increased complex I-driven respiration on its own (Figure 2D; blue open bar, see ^). Any concentration of Tat significantly impaired O_2_ consumption among AlloP- or morphine-treated cells (Figure 2D; hatched bars, see *), but AlloP was able to attenuate Tat impairment at the 100 nM concentration, and morphine was able to attenuate Tat impairment at all but the 600 nM concentration (Figure 2D; hatched bars, see ^). When respiration was driven by complex II, there was a notable rightward shift in the efficacy of Tat-mediated O_2_ consumption, consistent with the significant but modest effect previously observed (Figure 2E; hatched bars, see *). AlloP did not significantly alter complex II-mediated respiration, albeit morphine increased the potency of Tat, significantly shifting the Tat concentration–response curve to the left (Figure 2E; purple hatched bars, see *). Taken together, accumulation of Tat can substantially decrease O_2_ consumption, primarily via complex I, and AlloP or morphine may offset this effect. Tat’s effects at complex II are only observed at very high concentrations (≥500 nM) and may be modestly exacerbated by morphine. It is notable that O_2_ consumption was impaired when AlloP and morphine were co-administered together (see Supplemental Figure S2), consistent with our prior report of morphine exerting neurotoxic effects when combined with AlloP [21].

### 3.3. The Outer Mitochondrial Membrane Is Not Necessary for Tat-Mediated Impairment of Complexes I and II but May Influence Mitochondrial Interactions with AlloP

To extend the prior findings ex vivo and to further assess the important sites of action, mitoplasts (i.e., mitochondria without the outer membrane) were derived from the brains of C57BL/6N mice. Mitoplasts were exposed to a concentration–response gradient of Tat with or without pretreatment with AlloP or morphine as in the prior experiment (Figure 3A–C).

O_2_ consumption, driven by complex I, demonstrated a rightward shift in the mitoplast compared to that observed previously. Tat significantly reduced OCR at 500–800 nM concentrations (Figure 3D; yellow hatched bars, see *). However, morphine significantly shifted this effect leftward again, facilitating significant Tat-induced reductions in OCR at 200–800 nM (Figure 3D; purple hatched bars, see *). AlloP did appear to significantly change the effect of Tat on mitoplasts apart from a loss of efficacy for the Tat 600 nM concentration (Figure 3D; blue hatched bars, see *). When mitochondrial O_2_ consumption in the mitoplast was driven by complex II, the effect of Tat remained relatively insignificant at low concentrations, with significant OCR reductions observed at 500–800 nM concentrations (Figure 3E; yellow hatched bars, see *). However, morphine did not modulate this effect when driven by complex II (Figure 3E; morphine hatched bars, see *), and AlloP significantly increased overall OCR (Figure 3E; blue hatched bars, see ^), albeit without modulating the effective concentrations of Tat (Figure 3E; blue hatched bars, see *).

While it was unexpected that AlloP should exert a significant protective effect via complex II and not complex I, the proportional increase in OCR was similar across both experiments (14% increase via complex I and 17% increase via complex II). The lack of significant effect on complex I may be due to limitations in the dynamic range when driving OCR with malate/pyruvate vs. the faster rate achieved when driving with succinate.

### 3.4. Tat Impairs Respiration via Actions at Complexes I and II in Mouse Brain Mitochondria

To translate findings from cell culture to a whole animal model, Tat-tg mice that conditionally express a tat transgene [Tat(+) mice] or their non-tat-expressing counterparts [Tat(−) mice] were treated with AlloP (1 mg/kg) and/or acute morphine (30 mg/kg) before tissues were collected for mitochondrial preparation. Electron transport in isolated mitochondria was energized at complex I (Figure 4A,B) or complex II (Figure 4D,E) and assessed for OCR as previously described.

When the ETC was driven by complex I, Tat(+) mice demonstrated significantly reduced OCR in response to complex I or II activators compared to their Tat(−) counterparts (Figure 4C; see *). Commensurately, when the ETC was driven by complex II, Tat(+) mice also demonstrated a significant OCR deficit in complex II compared to Tat(−) controls (Figure 4E; see *). Pretreatment with AlloP in the absence (blue bars) or presence of morphine (red bars) fully attenuated these effects. No significant differences were observed in OCR for complexes III or IV, recapitulating the ETC complex targets identified in cellular experiments. Statistical comparisons were not made between complexes, but a notable apparent decrease was observed in complex IV of pharmacologically treated groups compared to control mice. Effects of Tat and AlloP are not thought to be influenced by gross changes in mitochondrial content, given that differences in the protein content of cytochrome *c* are not observed in the brain (Supplemental Figure S3).

### 3.5. AlloP or Morphine Attenuate the Generation of Tat-Mediated Intracellular Calcium and Reactive Oxygen Species

The functional effects for Tat to dysregulate [Ca^2+^]_i_ (Figure 5A–C) and to promote ROS formation (Figure 5D) were assessed in differentiated SH-SY5Y cells. The application of Tat precipitated a rapid influx of [Ca^2+^]_i_ that could be ameliorated by AlloP but not morphine (Figure 5A,B). A time-binned analysis demonstrated that treatment groups did not significantly differ prior to the administration of Tat (see arrow Figure 5A,B); however, Tat significantly increased [Ca^2+^]_i_ compared to control at all following time points, irrespective of morphine co-exposure (*p *< 0.05; Figure 5C; see *). Pretreatment with AlloP (10 nM) significantly attenuated the Tat-induced increase in [Ca^2+^]_i_ during the greatest Tat-promoted rises, which occurred between 30 and 600 s, irrespective of whether AlloP was co-administered with morphine (*p *< 0.05; Figure 5C; see ^). After Tat-induced [Ca^2+^]_i_ had fallen (660–1200 s), only the AlloP + morphine-treated cells remained significantly different from Tat or Tat + morphine treatment (*p *< 0.05; Figure 5C; see ^). Notably, even among AlloP-pretreated cells, there was a modest but apparent increase in [Ca^2+^]_i_ caused by Tat. However, this did not reach statistical significance until the last binned time point (660–1200 s; *p* = 0.05; Figure 5C; see *). Groups treated with AlloP + Tat did not differ from those treated with AlloP + Tat + morphine at any time point (Figure 5C).

Commensurately, Tat significantly increased the formation of ROS, as indicated by H_2_DCFDA, in differentiated SH-SY5Y cells compared to control cells (*p *< 0.05; Figure 5D; see *). Morphine or any concentration of AlloP significantly attenuated Tat’s capacity to drive ROS in these cells (*p *< 0.05; Figure 5D; see ^). It is notable that AlloP significantly reduced ROS formation on its own compared to controls either at the 10 nM concentration or when the 0.1 nM concentration was paired with morphine (*p *< 0.05; Figure 5D; see *).

Tat’s effects to promote ROS were further characterized in isolated, primary C57BL/6N striatal neurons (Figure 6A–C) and primary C57BL/6N mixed glia (Figure 6D). CellROX was used to differentiate the contributions of cytosolic vs. nuclear ROS in primary striatal mouse neurons (Figure 6A). Tat significantly induced cytosolic ROS with or without morphine present (Figure 6B; yellow bars; see *); however, nuclear ROS only tended to be significantly increased when Tat was administered (Figure 6C; yellow bars; *p* = 0.05). Irrespective of morphine, AlloP (10 nM) significantly attenuated cytosolic ROS (Figure 5B; blue bars; see ^). It is notable that morphine was not found to decrease cytosolic or nuclear ROS on its own, in contrast to what was seen in differentiated SH-SY5Y cells when ROS was detected with H_2_DCFDA (Figure 5D). However, this effect in SH-SY5Y cells was very small, and CellROX is known to express weak fluorescent signal, even in its reduced state, which may have limited its sensitivity in the present comparative analysis.

In primary mixed glia assessed by H_2_DCFDA, Tat significantly increased the formation of ROS compared to control cells (*p *< 0.05; Figure 6D; see *). Morphine or any concentration of AlloP significantly attenuated this effect (*p *< 0.05; Figure 6D; see ^). In particular, morphine or AlloP (10 nM) significantly decreased ROS formation on their own compared to the control (*p *< 0.05; Figure 6D; see *).

## 4. Discussion

Exposure to Tat inhibited mitochondrial respiration that was driven by complex I or complex II in a concentration-dependent manner. These findings were observed across models from permeabilized neuroblastoma cells to murine-derived mitoplasts, as well as mitochondria that were derived from mice exposed to Tat in vivo. Consistent with these findings, OCR has been found to be perturbed in complexes I and II among HIV-transgenic rats [30]; however, the transgenic HIV rat model also demonstrates perturbation of complex IV, which was not observed in the present work wherein mice were exposed to Tat alone. Acute exposure to a saturating concentration of morphine exerted no effect on mitochondrial OCR on its own; however, morphine modestly offset the complex-I-mediated effects of Tat in neuroblastoma cells and the murine mitoplast. It is notable that morphine did not exert these protective effects when acutely administered in vivo*,* which could be due to pharmacodynamic differences when translating from cell culture to a whole-animal model. Unlike morphine, AlloP facilitated the efficiency of electron transfer and the resulting OCR when administered alone in neuroblastoma cells (complex I) and the mitoplast (complex II). When co-administered with Tat exposure, AlloP attenuated the Tat-mediated impairment of electron flow in neuroblastoma cells and fully ameliorated Tat-mediated disruption of complexes I and II in vivo. However, AlloP and/or morphine caused an apparent reduction in ASC/TMPD-driven respiration in both Tat(−) or Tat(+) mice. When the influx of [Ca^2+^]_i_ and the generation of ROSwere assessed, physiological concentrations of AlloP were found to attenuate Tat-mediated effects, irrespective of pretreatment with morphine. Taken together, these results suggest that HIV-1 Tat compromises mitochondrial function through actions involving respiratory complexes I and II and that physiological AlloP may exert protective effects. These data implicate several potential molecular mechanisms for future investigation.

Mitochondria are well-described targets of viruses, including HIV. In general, viruses can modulate mitochondrial function to promote their own replication, escape host immune responses, and facilitate virion escape from a host cell, often by promoting apoptosis [62,63]. Such effects may have been selected for based on the needs of the viral life cycle, with viruses tending to hyperpolarize mitochondrial membranes early in infection to promote cell survival/immune escape and dissipating mitochondrial membrane potential later in infection to promote apoptosis and the release of viral progeny [62]. While multiple HIV virotoxins are likely to act in concert to modulate mitochondrial function, the current report focuses on those that are Tat-mediated and thus potentially active even in virally-suppressed PLWH. Upon sustained exposure, Tat may contribute to the well-described effects on mitochondrial morphology, biogenesis, fusion, fission, and microtubule transport that are observed in HIV [64]. Using Tat-transfected cells, Tat has been observed to depolarize mitochondrial membranes, generate ROS, and activate pro-apoptotic Caspace-3 [65]. In a prior report, the loss of mitochondrial membrane potential occurred concurrent with the nearly exclusive accumulation of Tat within mitochondria [65]. These data demonstrate Tat’s likely involvement in mitochondrial-mediated cell death; however, the mechanisms by which Tat initiates mitochondrial dysfunction, presumably preceding gross changes in morphology, are poorly understood but may be more susceptible to rescue by therapeutic intervention.

High concentrations of Tat have been studied, and the effects on mitochondria have been found to be independent of direct actions to open the mPTP or to activate the canonical Bax/Bak-mitochondrial permeabilization pathway [66]. A study using supraphysiological Tat concentrations found that the anion transport inhibitor, DIDS, could block Tat-mediated reduction in OCR [66]. Similarly, recent investigations using more physiological concentrations of Tat with SH-SY5Y neuroblastoma cells find proteomic downregulation of voltage-dependent anion channels (VDAC) [19], which are targets of DIDS. VDAC and the mitochondrial Ca^2+^ uniporter (MCU), an electrogenic uniport, are the primary sources for Ca^2+^ transport into the outer and inner mitochondrial membranes, respectively. VDAC is proposed to be one of the proteins that comprises the mPTP and regulates Ca^2+^ influx at the outer mitochondrial membrane. The MCU is responsible for transporting Ca^2+^ across inner mitochondrial membrane, driven by the negative charge of the mitochondrial membrane potential (MMP). Thus, a fall in MMP alters the Ca^2+^ influx via the MCU [67]. Using more physiological concentrations of Tat and an animal model that expresses CNS *tat* copy numbers thought to be consistent with cART-suppression, we have found Tat to depolarize mitochondrial membrane potential and increase intracellular Ca^2+^ [21,32,59]. While the mechanisms for Ca^2+^ flux were not probed in those studies, others have found that calcineurin, a phosphatase activated by Ca^2+^ that regulates the translocation of Parkin and downstream mitophagy [68], is critical in the early phase of Tat-mediated morphological dysfunction [64]. Additional sources of Ca^2+^ may also involve release from endoplasmic reticulum stores. Mitochondria–endoplasmic reticulum cross-talk occurs at the mitochondria-associated membranes (MAMs), and ion exchange occurs between these organelles. The IP3 receptor serves as a channel to flux Ca^2+^ from the endoplasmic reticulum to mitochondria via VDAC. Recent work demonstrates the capacity of Tat to downregulate tethering proteins that comprise the MAM, including VDACs and IP3 receptors [69] and herein, we find that Tat rapidly drives [Ca^2+^]_i_ influx and the generation of ROS. Others find Tat to reduce the maximal and spare respiratory capacity of mitochondria in addition to reduced ATP synthesis and increased Ca^2+^ and ROS [19]. These data support the notion that Ca^2+^-dysregulation is a critical trigger for the early phase disruption of electron transfer, formation of ROS, and the signaling cascade that promotes Tat-mediated mitochondrial dysfunction and mitophagy.

Opioids may exert additive effects to promote Tat-mediated CNS dysfunction, albeit the relationship between these factors is complex, and additivity can involve separate, non-interacting mechanisms such as those demonstrated to drive the recruitment of myeloid cells in the CNS [10]. Certainly, glia drive a great proportion of the morphine/Tat interactions found to promote neurotoxicity and the present findings are consistent with these observations, revealing no Tat/morphine interaction on cytosolic ROS when modeled in isolated primary neurons (Figure 5A,B). However, it is intriguing that ROS was only significantly elevated when morphine was included with Tat (Figure 5C). While an estimated 90% of ROS is generated by electron escape along the ETC, cyclooxygenases in the nucleus also generate ROS (in addition to other cellular sources) [70] and morphine may stimulate expression of cyclooxygenases [71,72]. Chronic morphine has also been associated with mitochondrial damage and the generation of ROS, and this is associated with its capacity to promote mitochondrial dysfunction while inhibiting mitophagy [73], the latter occurring via suppression of Parkin/Pink1 recruitment to damaged mitochondria [73]. For mitophagy to occur, Parkin must be recruited to the damaged mitochondria, and such recruitment is dependent on Pink1 accumulation in the outer mitochondrial membrane. Morphine inhibits the phosphorylation of Parkin S65A, which is necessary for the ubiquitination of mitochondrial proteins upon loss of membrane potential [73]. Morphine further inhibits the lysosomal fusion of mitochondria after they are damaged by rotenone and can promote the degradation of Pink1 [73]. As such, the apparent protection that morphine exerted in the present work by shifting Tat’s effects on OCR to the left (Figure 2D) may not necessarily indicate healthier mitochondria. Rather, morphine may promote inefficient and leaky electron transport in SH-SY5Y mitochondria. When the outer mitochondrial membrane (OMM) was removed, morphine’s apparent protective effects were attenuated (see mitoplasts in Figure 3), implicating the importance of OMM proteins in this effect.

Mitoprotective agents, such as AlloP, may have utility in the treatment of neuroHIV. We have reported several mechanisms by which AlloP may offset Tat-mediated neurotoxicity, including its capacity to potentiate GABA_A_-mediated Cl^−^ influx and inhibition of L-type voltage-gated Ca^2+^ channels, either of which may attenuate excitotoxic insult [32]. However, AlloP-mediated effects on mitochondria are less well-studied. There are reports of AlloP regulating the opening of the mPTP and increasing recruitment of Bcl-2 protein expression, which may be involved [33,34,35,36,37,38]. In particular, Bcl-2 is an integral OMM protein for cell survival [74,75] and regulates the opening of VDAC on the OMM [76]. It is notable that the protective effects of AlloP to raise OCR on its own or to offset Tat’s effects to reduce OCR were lost when the OMM was removed (Figure 3). We have also found AlloP to prevent Tat-induced loss of mitochondrial membrane potential and herein we find it to also be efficacious in reducing Tat-mediated Ca^2+^ influx and ROS production. Bcl-2 is involved in restoring basal mitochondrial membrane polarization states and OCR [77], regulating the generation of ROS, and triggering the release of cytochrome *c* [78]. Given that the Bcl-2 protein is localized to OMM [79], it may have contributed to the AlloP-mediated protection observed. The mitoprotective effects of AlloP may also exert an indirect benefit by sparing mitochondria and their own antiviremic actions. The mitochondrial antiviral-signaling protein (MAVS) may be important for the formation of the NLRP3 inflammasome in response to viral infection [78]. The MAVS induces signaling that recruits innate immune factors, including interferons and NF-κB. Stimulators of interferon genes (STING) can also bind MAVS within the MAM, and the two are proposed to exert an additive effect in mounting a greater antiviral innate immune response [80,81,82].

## 5. Conclusions

Taken together and in consideration of the extant literature, these data highlight the critical role that Tat-driven [Ca^2+^]_i_ plays in HIV modulation of the mitochondria, likely as an early or inciting event to indirectly modulate electron transfer across the ETC and thereby exerting downstream effects to drive ROS formation, deplete ATP, and ultimately prompt cellular apoptosis when it is advantageous. Morphine can suppress mitophagy, appearing as a protective effect in some contexts but potentially allowing viral modulation of mitochondria to continue. We find ETC complexes I and II as primary targets of disruption by a physiological concentration of Tat, translated from in vitro to ex vivo models. AlloP exerted mitoprotective effects, increasing OCR on its own and attenuating Tat-mediated reductions in OCR both in cell culture and in ex vivo mitochondria purified from Tat-exposed mice. AlloP-mediated protection appeared dependent on OMM-protein interactions, perhaps implicating actions at Bcl-2 or VDACs in addition to its known extra-organellar effects to offset excitotoxicity. Taken together, Tat may contribute to mitochondrial dysfunction in the CNS of PLWH, even those that achieve viral suppression, and anti-Tat therapeutics such as AlloP may exert beneficial effects. Future work will aim to identify the important OMM proteins that mediate AlloP effects and to assess their influence on cell populations that are selectively vulnerable to HIV insult.

## Data Availability

Data are available upon request.

## Supplementary Materials

The following supporting information can be downloaded at https://www.mdpi.com/article/10.3390/antiox14040420/s1. Supplemental Methods and Supplemental Figures S1–S3.

## Abbreviations

The following abbreviations are used in this manuscript:

AlloP	Allopregnanolone
cART	Combination antiretroviral therapy
DIDS	4,4′-Diisothiocyano-2,2′-stilbenedisulfonic acid
HAND	HIV-associated neurocognitive disorders
mPTP	Mitochondrial permeability transition pore
OCR	Oxygen consumption rate
OMM	Outer mitochondrial membrane
PLWH	People living with HIV
VDAC	Voltage-dependent anion channels
Tat	Trans-activator of transcription
Tat-tg	HIV-1 *tat*_1–86_ transgenic
