# Supplementary material for: HIV-1 Tat Impairment of Mitochondrial Respiration via Complexes I and II Can Be Ameliorated by Allopregnanolone in Opioid-Exposed or Opioid-Naïve Cells and Mice
Source: Antioxidants (Basel). 2025 Mar 31;14(4):420. doi: 10.3390/antiox14040420 (PMC12023989; doi:10.3390/antiox14040420)
Supplement: Supplementary file 1 [file antioxidants-14-00420-s001.zip › antioxidants-3419485-supplementary.pdf]

SMahdi, F.; Shariat-Madar, Z.; Paris, J.J. HIV-1 Tat Impairment of Mitochondrial Respiration via Complexes I and II Can Be Ameliorated by Allopregnanolone in Opioid-Exposed or Opioid-Naïve Cells and Mice. Antioxidants 2025, 14.

Supplemental Methods

Western blot

Snap-frozen HIV-1 Tat(−) transgenic and HIV-1 Tat(+) mice brains were suspended in 1X cold RIPA sterile-filtered buffer (20 mM Tris-HCl pH 7.5, 150 mM NaCl, 5 mM EDTA, 1% NP-40, 1% sodium deoxycholate, 0.1% SDS) containing Halt protease inhibitor cocktail (Thermo Fisher). Brains were homogenized in 0.5 mL cold RIPA buffer for every 10 mg of tissue. The homogenate was centrifuged at 10,000× *g* for 30 min at 4 C. Protein concentration was determined. Protein samples (100 µg) were separated on SDS-PAGE 4–20% gel (Bio-Rad, Hercules, CA, USA) and transferred to 0.45 µm nitrocellulose membranes. The membranes were blocked with Odyssey® Blocking Buffer in TBS (LI-COR, Inc., Lincoln, NJ, USA) for 1hr at room temperature. The membranes were then incubated with primary antibodies to cytochrome *c* at 0.5 µg/mL and GAPDH as loading control at 1:500 dilution (Santa Cruz Biotechnology, Inc., Dallas, TX, USA) in blocking buffer with gentle agitation at 4 °C overnight. The membranes were washed with TBST four times for 15 min each time. After washing, the membranes were incubated with an IRDye® 800CW Goat anti-mouse against cytochrome *c* and IRDye® 680RD donkey anti-goat against GAPDH, all at 1:4000 in blocking buffer for 1 h at room temperature. After three washes with TBST, the electroblotted proteins were detected with LI-COR Odyssey CLX to visualize the protein bands. Relative band intensity was quantified using ImageJ software [1].

Reference

1. Schindelin, J.; Arganda-Carreras, I.; Frise, E.; Kaynig, V.; Longair, M.; Pietzsch, T.; Preibisch, S.; Rueden, C.; Saalfeld, S.; Schmid, B.; et al. Fiji: An open-source platform for biological-image analysis. *Nat. Methods* **2012**, *9*, 676–682.

Supplemental Figure S1

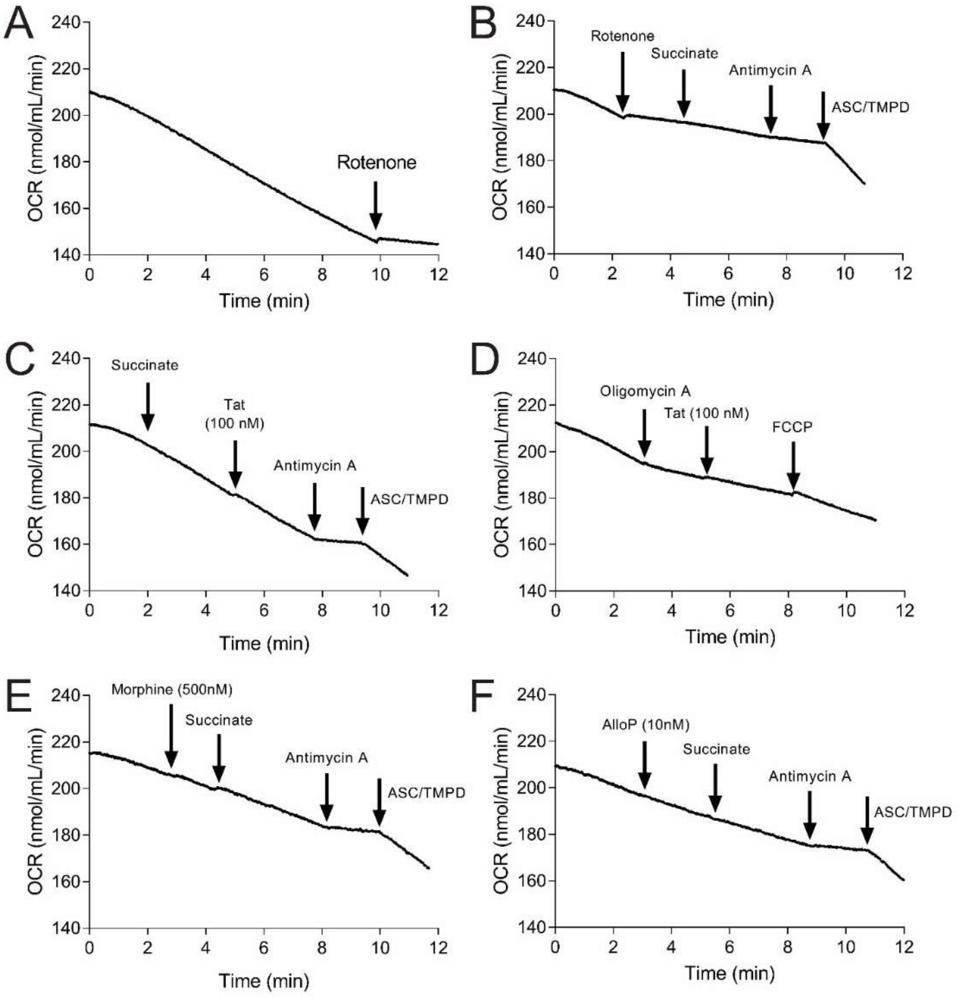

Figure S1. (A) Oxygen consumption rate (OCR) of permeabilized SH-SY5Y human neuroblastoma cells were assessed via Clark-type electrode. Respiratory response of mitochondria and electron transport chain complexes was confirmed via exposure to (A) an inhibitor of complex I (rotentone), (B) sequential exposure to rotenone, an activator of complex II (succinate), an inhibitor of complex III (antimycin A), and activators of complex IV (ascorbate & TMPD). The continued responsiveness of complexes exposed to HIV Tat protein was confirmed with (C) complex-II-driven Tat exposure followed by inhibition of complex III and activation of complex IV and (D) complex-III-driven Tat exposure followed by uncoupling of oxidative phosphorylation via FCCP. The respiratory response of mitochondria following (E) morphine or (F) allopregnanolone (AlloP) was also confirmed.

Supplemental Figure S2

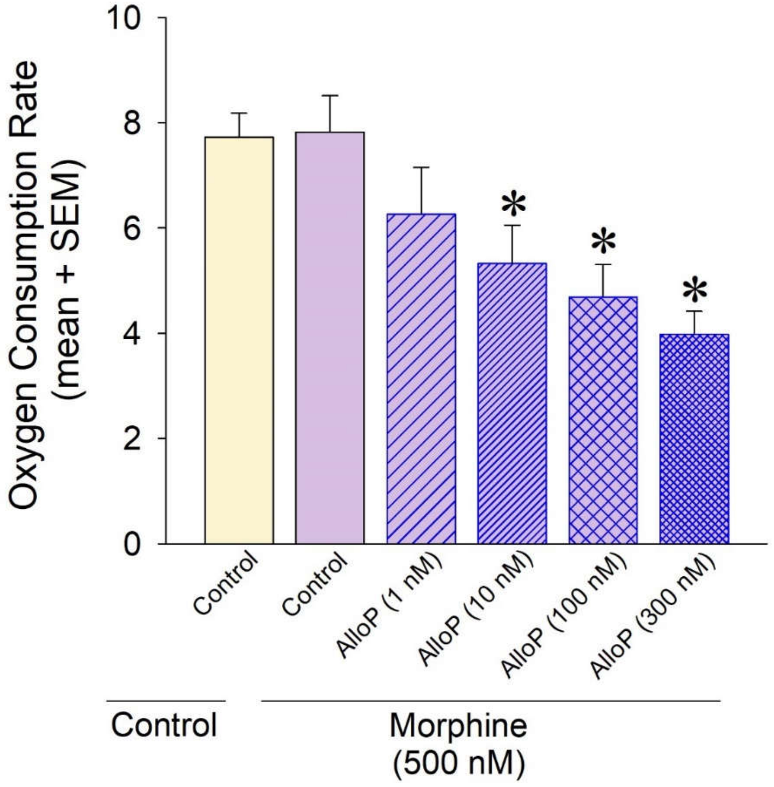

Figure S2. Allopregnanolone (AlloP) and morphine interacted to reduce the oxygen consumption rate of permeabilized SH-SY5Y human neuroblastoma cells in a concentration-dependent manner (n=4 independent cultures/group) [$F(5,18)=10.07$, $p<0.05$]. *indicates significant difference from control (yellow bar; one-way ANOVA, $p < 0.05$).

Supplemental Figure S3

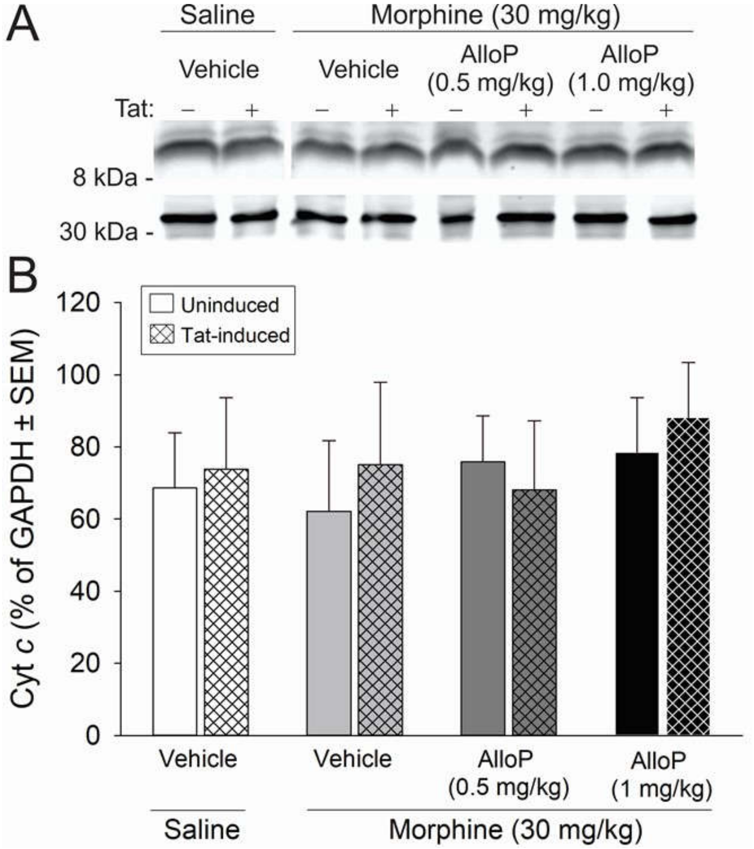

Figure S3. Western blot demonstrating no significant difference in whole-brain cytochrome *c* (Cyt *c*) protein content among Tat(–) or Tat(+) transgenic mice exposed to an acute administration of morphine (i.p.) or subchronic exposure to allopregnanolone (AlloP; QD for 7 days, s.c.).
